# Supplementary material for: HIVprotI: an integrated web based platform for prediction and design of HIV proteins inhibitors
Source: J Cheminform. 2018 Mar 9;10:12. doi: 10.1186/s13321-018-0266-y (PMC5845081; doi:10.1186/s13321-018-0266-y)
Supplement: Supplementary file 2 — Additional file 2. Source code of HIVProtI web server. [file 13321_2018_266_MOESM2_ESM.zip › HIVprotI_Source-code/hivprotI/weka-3-6-6/doc/weka/gui/visualize/VisualizeUtils.html]

VisualizeUtils


---


|  |  |  |
| --- | --- | --- |
| **Overview** \| **Package** \| **Class** \| **Tree** \| **Deprecated** \| **Index** \| **Help** \| **Weka's home** | |  |
| **PREV CLASS**   NEXT CLASS | **FRAMES**    **NO FRAMES**     **All Classes** |  |
| SUMMARY: NESTED \| FIELD \| CONSTR \| METHOD | DETAIL: FIELD \| CONSTR \| METHOD |  |


---


## weka.gui.visualize Class VisualizeUtils

```
java.lang.Object
  weka.gui.visualize.VisualizeUtils
```

---

``` public class VisualizeUtils extends java.lang.Object ```

This class contains utility routines for visualization

**Version:**
:   $Revision: 7059 $

**Author:**
:   Mark Hall (mhall@cs.waikato.ac.nz)

---

**Constructor Summary**

`VisualizeUtils()`


| **Method Summary** | |
| --- | --- |
| `static java.awt.Color` | `processColour(java.lang.String colourDef, java.awt.Color defaultColour)`             Parses a string containing either a named colour or r,g,b values. |

**Methods inherited from class java.lang.Object**

`equals, getClass, hashCode, notify, notifyAll, toString, wait, wait, wait`

**Constructor Detail**

### VisualizeUtils

```
public VisualizeUtils()
```


**Method Detail**

### processColour

```
public static java.awt.Color processColour(java.lang.String colourDef,
                                           java.awt.Color defaultColour)
```

:   Parses a string containing either a named colour or r,g,b values.

    :   **Parameters:**: `colourDef` - the string containing the named colour (or r,g,b): `defaultColour` - the colour to return if parsing fails **Returns:**: the Color corresponding to the string.


---


|  |  |  |
| --- | --- | --- |
| **Overview** \| **Package** \| **Class** \| **Tree** \| **Deprecated** \| **Index** \| **Help** \| **Weka's home** | |  |
| **PREV CLASS**   NEXT CLASS | **FRAMES**    **NO FRAMES**     **All Classes** |  |
| SUMMARY: NESTED \| FIELD \| CONSTR \| METHOD | DETAIL: FIELD \| CONSTR \| METHOD |  |


---
